# The Influence of the Parameters of the Skew Rolling Process for Bimetallic Elements on the Mechanical Properties and Structure of Materials

**DOI:** 10.3390/ma17184558

**Published:** 2024-09-17

**Authors:** Tomasz Kusiak, Janusz Tomczak, Jarosław Wójcik

**Affiliations:** Department of Metal Forming, Mechanical Engineering Faculty, Lublin University of Technology, 20-618 Lublin, Poland; j.tomczak@pollub.pl (J.T.); jaroslaw.wojcik@pollub.pl (J.W.)

**Keywords:** skew rolling, bimetal rolling, FEM, welding, microstructure, mechanical properties

## Abstract

This paper presents selected results of theoretical and experimental research into the manufacture of axisymmetric bimetallic components using three-tool skew rolling technology. In the tests, it was assumed that the outer layer would be a material intended for heat treatment. However, low-carbon steel was used for the core. Experimental investigations were carried out in an innovative CNC skew rolling mill. Tests were carried out at different technological parameters of the process. In addition, the geometric parameters of the billet and the way it was heated were analyzed in relation to the quality of the resulting weld between the two materials. The quality of the weld was assessed based on metallographic observation and on strength tests (shear method). On the other hand, theoretical studies were based on numerical modeling (FEM). The numerical analysis made it possible to determine the distribution of temperature, deformation and stress in the rolling bimetallic component. The results obtained indicated that it is possible to produce bimetallic materials from the proposed steel grades. In addition, a significant effect of the method of heating the billet in the chamber furnace on the microstructure in the joining zone and the shear strength was found. There was an increase in Rc strength of about 35% when using oxidation protection. The results indicated better strength when the billet is rolling with a smaller outer layer thickness (about 50 MPa). This was confirmed by the results obtained from the FEM analysis, which indicated higher values of plastic strain and the occurrence of higher compressive stresses in the near-surface zones of the rolled bimetallic forging, both of which facilitate the welding process. From the temperature distribution (in the range of (600–1200) °C) obtained during the rolling of the bimetal forging, it can be seen that contact with cold tools does not affect the temperature in the welding zone.

## 1. Introduction

In recent times, there have been increasing demands for materials used in the construction of machinery and equipment, which are characterized by unique mechanical and physicochemical properties. This is contributing to an intense interest in bimetallic materials [1]. A bimetallic material is characterized by a permanent joint over the entire contact surface of two metals or metal alloys. The quality of the joint is extremely important, affecting the correct operation of the bimetallic component [2]. The joint of two different metals or metal alloys results in a bimetal having the constituent characteristics of these materials. Therefore, it is important to select the right metal layers when designing machine parts.

For example, corrosion-resistant coatings are applied to produce bimetallic material operating in corrosive environments. Such a solution has many advantages in nullifying the risk of corrosion, and it is also economically advantageous due to the cost of the bimetallic material compared to a homogeneous stainless steel part [3]. The use of a corrosion-resistant protective layer is extremely important in the aerospace, automotive and railway industries, where the weight of the part is important, affecting environmental factors by reducing fuel consumption and therefore reducing CO2 emissions [4,5,6]. The solution is bimetallic parts made of lightweight magnesium alloys, which are susceptible to corrosion, creating the need for a coating of corrosion-resistant aluminum alloy [7]. This type of research was conducted by Mróz et al., who focused on the feasibility of manufacturing by the explosion method a bimetallic rod made of 1050A and AZ31 materials [8]. Subsequently, in a subsequent study by Mróz et al., the bimetallic rod thus produced was subjected to the process of metal forming again by rolling. This improves the mechanical properties by modifying the microstructure and allows the bimetal to be shaped with an optimized geometry [9]. Undoubtedly, explosion bonding is a popular method for manufacturing bimetallic materials; however, this technology has disadvantages, among which can be included the need for explosives, a suitable site (training ground) and considerable time to prepare the feedstock. In addition, the technology of producing bimetals using explosive energy has mainly been extensively studied for the production of flat bimetal components, i.e., sheet metal [10].

Mróz et al. also analyzed the feasibility of making a Mg/Al bimetallic handle for aerospace applications, which was produced by the die forging method from a rod previously produced by the explosive method. The use of die forging produced forgings with better mechanical properties than the feedstock materials. However, it was found that there was a problem with the loss of the aluminum alloy cladding layer during the forging process, which was the result of too high a heating temperature. This phenomenon exacerbates the differences in the yield strength of the different bimetallic components. The quality of the bimetallic joint was observed to be highest in the areas with the smallest values of deformation strain [11]. Forming a bimetal by metal forming, most commonly die forging, leads to the formation of a uniform bimetallic part. Unfortunately, as studies have shown, this process is very complicated and expensive; moreover, it does not guarantee the production of a correct joint. In addition, large presses with high pressures are required, which affects tool life and significantly increases the energy intensity of the process.

Chugreeva et al. [12] have successfully attempted to make a bimetallic gear by die forging from a previously prepared batch by PTA welding. They used 41Cr4 for the outer layer and C22.8 for the core. This type of combination of two materials provides another of the decisive factors in the use of bimetallic material, which is the improvement in strength properties that increase the durability of working machine parts. In this case, the outer layer is a more abrasion-resistant material for highly stressed parts, while the core material is made of steel with high ductility and good impact resistance. Such a solution is used in the production of axle shafts, where it is often required that the outer layer is strong and abrasion-resistant, while the core is ductile.

The die forging method can also be used to combine different materials, e.g., aluminum alloys and steel; such a combination results in a reduction in the weight of the components. Wohletz and Groche focused on evaluating the effect of the forging temperature of aluminum alloy and steel on the quality of the resulting weld. Higher temperatures resulted in an increase in the quality of the joint, and the plastic yielding of the material was more similar [13]. Similar studies on the quality of the aluminum–steel joint were conducted by Chen et al. [14]; in their work, the effect of plastic strain was determined. It was noted that higher values of strain resulted in the formation of microcracks at the interface. Research was also conducted in which bimetallic material was produced by rolling using a thixotropic core [15]. The use of a thixotropic core minimizes the risk of such material defects as occur during casting, i.e., porosity and voids. However, it does not fully eliminate the risk of such defects. Ossenkemper et al. [16] conducted research on cold forging of bimetallic components. However, this type of technology only allows the formation of a very weak bond between two materials, based mainly on mechanical interlocking.

Numerous studies are being conducted on the possibility of combining different grades of materials. For example, the use of a combination of steel and titanium [17], titanium and aluminum alloy [18] or aluminum alloy and copper [19] leads not only to a reduction in the weight of these components but also to the optimization of production costs, and it also allows a reduction in the use of alloying elements. However, such material combinations often require special manufacturing processes, e.g., rolling with a thixotropic core or manufacturing bimetals by explosive methods.

Another of the methods of manufacturing bimetallic materials is cross-wedge rolling. Studies of the manufacture of 42CrMo/Q235 bimetallic material were conducted by Longfei Lin et al. [20]. They noted that the quality of the joint was extremely significantly affected by ovalization during rolling. In addition, there was a risk of material cracking. This is due to the characteristic state of stress during rolling due to the resulting Mannesmann effect [21]. In addition, cross-wedge rolling technology requires expensive tools, often designed for a specific product. Therefore, manufacturing bimetallic material with this technology is more cost-effective for high-volume production. A similar study was conducted by the authors Zhu et al. [22]. They determined the effect of heat treatment on the quality of the joint of bimetallic parts made by cross-wedge rolling technology.

There has been extensive research on casting bimetallic materials. For example, such research has been conducted by Simsir et al. [23]. The authors analyzed the casting process of 316L/30CrNiMo8 bimetal and its effect on the quality of the weld. The casting of bimetals often leads to a durable weld, but there is a risk of casting defects. A study by Ji et al. [24] provided interesting information. The authors used an innovative method of manufacturing bimetallic tubes using CLCRB casting technology. This involves applying a liquid material to a solid material. The research confirmed the risk of the formation of brittle phases during this process due to the oxidation of the bonded surfaces.

A typical and quite popular method of joining two materials is friction welding. In the article, Ma et al. analyzed the process of friction-welding carbon steel to stainless steel [25]. Friction welding often leads to a permanent connection. However, it is mainly possible to make a butt weld between two surfaces. With friction welding, it is not possible to make a bimetallic rod with different materials in the outer layer and core.

This paper presents only some possibilities for welding different metal types and their influence on the mechanical and physicochemical properties of bimetals. In view of this, it can be concluded that bimetallic material has a wide range of applications in industries including the aerospace, automotive, marine, chemical, electrical engineering, textile and food industries, among others [26,27,28]. Such a wide application of bimetallic materials is causing the technology of their manufacture to develop intensively, and new effective methods for their production are constantly being sought.

One of the alternative methods of manufacturing bimetallic materials is three-tool skew rolling in an innovative numerically controlled (CNC) skew rolling mill. The first trials of skew rolling of bimetallic rods have been carried out using combinations of different grades of steel, i.e., stainless steel–unalloyed steel and unalloyed steel–medium alloyed steel combinations [29]. The results confirmed the feasibility of using bevel rolling to produce bimetallic rods. The use of CNC rolling mills has many advantages: among others, it not only enables the forming of bimetallic rods but also makes it possible to form forgings with variable cross-sections of various types of stepped shafts and axles, as well as pre-forgings that can serve as billets in other metal forming processes [30]. In addition, it is possible to roll unit parts with one set of tools without replacing them. The unquestionable advantages of rolling are the high efficiency of the process and the introduction of low forces, which significantly affects the energy efficiency of the process. In addition, the stresses during skew rolling with three tools do not cause the risk of material defects in the form of cracks [31]. In view of the undoubted advantages of three-tool skew rolling, a study was conducted for the production of a bimetallic rod consisting of an outer layer of load- and wear-resistant steel over a core with an increased yield strength. Thus, the main purpose of the study was to determine the effects of the parameters of the skew rolling process on the quality of the resulting welded joint of two different grades of steel (C60/S355). It should be noted that the results presented in this paper are a prelude to a broader scientific study of the manufacture and forming of bimetallic products in a CNC skew rolling mill, where attempts will be made to combine different steels and their alloys.

## 2. Materials and Methods

This article presents the results of research on the skew rolling process of C60/S355 bimetallic materials. The analysis of the test results was based on microscopic examination (analysis of the microstructure in the interface zone with determination of the average grain size) then the strength parameters of the resulting joint (wall strength) were determined. In addition, an analysis of force parameters was carried out; these parameters are important from an economic point of view and affect tool wear as well. Additionally, a numerical analysis was performed using the finite element method for skew rolling the bimetal with the same parameters as during the experimental tests.

**Figure 1:**
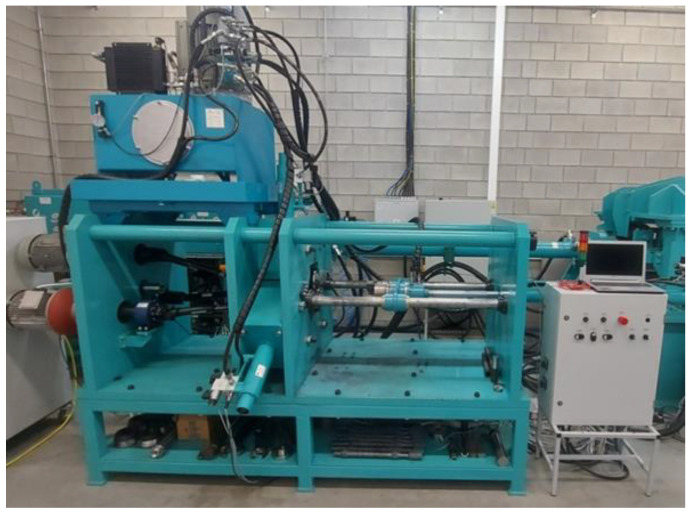
CNC skew rolling mill.

The experimental research was carried out using an innovative numerically controlled (CNC) skew rolling mill designed at the Lublin University of Technology (Figure 1). The innovation of the machine is that, using a single set of tools, it is possible to form an arbitrarily stepped axisymmetric element, by introducing an appropriate program controlling the tools and the chuck. Thus, it can be concluded that the technological potential of the machine is very high, with relatively low production costs.

### 2.1. Materials Used for Research

Given the advantages of the innovative CNC skew rolling mill, eight different variants of the test for the possibility of combining two steels by skew rolling technology were analyzed. The material used as the outer layer of the bimetal was a tube of medium carbon steel for heat treatment in C60 grade. However, the core was made of S355J structural steel. Table 1 shows the chemical composition of the materials used in the study, sourced from the domestic market.

### 2.2. Process Parameters

**Figure 2:**
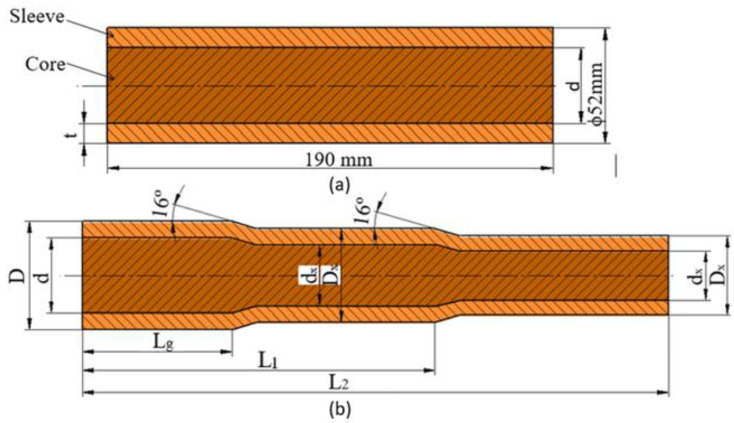
Designation of the dimensions of the starting material and the dimensions of the rolling material: (**a**) billet; (**b**) bimetallic forging.

The analysis on the quality of the weld was carried out on the influence of such parameters as the geometric parameters of the billet (sleeve wall thickness t), the rolling parameters and the influence of fusion welding of the front surfaces of the starting material were analyzed. To simplify the process and analyze a larger number of parameters, the rolling process on one billet of a two-step forging was adopted (Figure 2b). In this way, it is possible to determine at the same time the influence not only of the initial billet outer layer thickness (t) but also of the diameter reduction value δ described by Equation (1).

δ = D/D_x_ (1)

where D = diameter before rolling and Dx = diameter after rolling.

Figure 2a shows the dimension designations of the starting material (billet). For variants I, III, V and VII, a diameter reduction ratio of δ = 1.14 was used and a rolling velocity (chuck movement velocity) of Vc = 15 mm/s was assumed, and for the other variants δ = 1.27 and Vc = 25 mm/s, respectively. The kinematic parameters of the process were assumed for the tests (the rotational speed of the tools, the forward speed of the rollers and the axial speed of the rolled billet), consistent with the values for skew rolling of solid products, which were analyzed in studies on this subject [32]. The selection of progressive velocities of the rolled material was also guided by the flow kinematics of the material, so that the sum of the resultant velocities associated with the elongation of the material and the twisting of the shaft axes would be close to the speed of the chuck with the billet.

In all variants of the test, the billet length was 190 mm and the outer diameter was D = 52 mm. In contrast, the initial core diameter (d) varied in valence from the variant adopted. In variants I, II, V and VI it was ϕ 38 mm, in the other variants ϕ 34 mm. In contrast, the outer layer thickness (t) of the billet was 7 mm and 9 mm. The percentage of the outer layer made of C60 steel was 46.5% and 57.3%, respectively. Due to the risk of decarburization of the combined layers during heating of the billet material in the chamber furnace, the effect of pre-bonding of the front surfaces in the billet was analyzed in the last four variants, i.e., V–VIII, using the fusion welding technology. Table 2 shows the input factors for the rolling process of bimetallic materials. The shape and dimensions of the bimetallic material forging are shown in Figure 2b. Due to the specific features of the rolling process, it was assumed that the workpiece material would not be rolled in the chuck zone. However, under industrial conditions, it is possible to carry out rolling without any allowance for the chuck zone. The length of the chuck zone was assumed to be l_g_ = 80 mm. The other lengths of the bimetal forging were assumed so that the necessary analysis of mechanical strength properties and macro- and microscopic observations could be made. The length measured from the beginning of the forging to the end of the first step was l_1_ = 150 mm, and the length of the entire forging was l_2_ = 250 mm.

**Figure 3:**
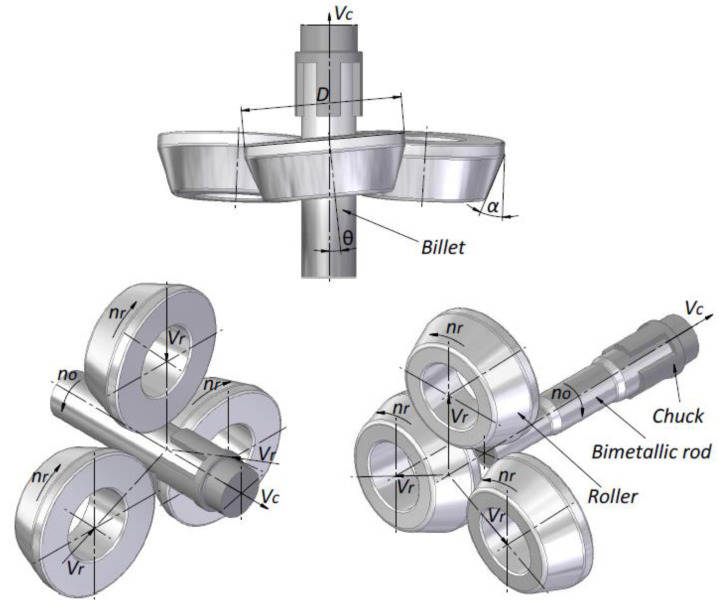
Schematic diagram of skew rolling (description in text).

**Figure 4:**
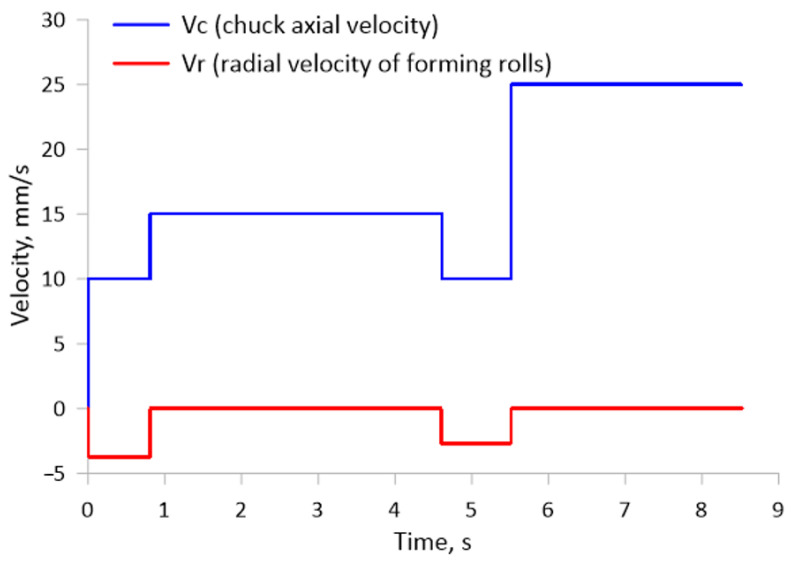
Velocity diagram of forming tools and chuck during skew rolling of bimetallic material.

A diagram of skew rolling in a CNC rolling mill is shown in Figure 3. Skew rolling of bimetallic rods is carried out with three tools arranged symmetrically around the rolling axis every 120°. Each tool has an adjustable skew angle, relative to the rolling axis (ϴ), of −10° to +10°. In the tests of rolling the bimetallic material, the tool skew angle ϴ was assumed to be 5°. The tools are tapered cylinders, where a cylindrical surface with a diameter of Dr = 150 mm is used to calibrate the shaped part. The forming part, on the other hand, is a tapered surface with an angle of inclination α (α = 20° was assumed in the tests). The tools perform rotary motion in the same direction at a constant rotational speed of no = 60 rpm with simultaneous numerically controlled movement of the rolling material in the direction of the rolling axis at a velocity of Vr. The correct choice of tool radial velocity (Vr) and chuck axial velocity with material (Vc) results in the desired dimensions of the bimetallic forging. In the tests, the tools performed radial movement towards the axis of the bimetallic forging at a velocity of 2.7 mm/s and 3.75 mm/s (depending on the product step being formed). For the chuck, an axial velocity of 10, 15 or 25 mm/s was used, depending on the step being shaped. The exact time course of the individual velocities is shown in Figure 4.

### 2.3. Metallographic Analysis

In order to analyze the microstructure in the interface zone of two materials, samples were cut from the bimetallic material obtained after skew rolling, as well as from the core and tube materials not subjected to the rolling process. The samples were cut in cross-section. Mounting was performed so that the samples were stably fixed in the resin and could be subjected to further processing. The samples were then ground and polished. The microstructure of the obtained samples was revealed by adding 3% Nital etchant on the surface of the samples for about 15 s. Observations were performed using a Keyence VHX-7000 digital optical microscope with a magnification of 700×.

## 3. Experimental Research

**Figure 5:**
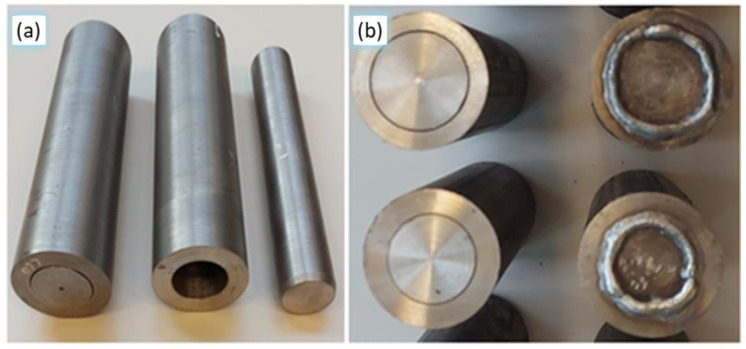
Billet prepared for rolling: (**a**) joint C60/S355 material in H7/p6 tolerance; (**b**) billet front surfaces—unwelded and fusion-welded.

Before the rolling process in the CNC skew rolling mill, the billet was properly prepared. The inner surfaces of the tube as well as the outer surfaces of the core were machined to tolerance. In the tests, a tight fit was assumed between the tube and the core to a tolerance of H7/p6. In this way, the machined surfaces were cleaned of oils and other impurities. The tube and core were then initially joined together (Figure 5a). In the last four variants (V–VIII), the billet front surfaces were additionally pre-joined by fusion welding (Figure 5b). The billets thus prepared were heated in a chamber furnace to a homogeneous hot rolling temperature of 1180 °C.

**Figure 6:**
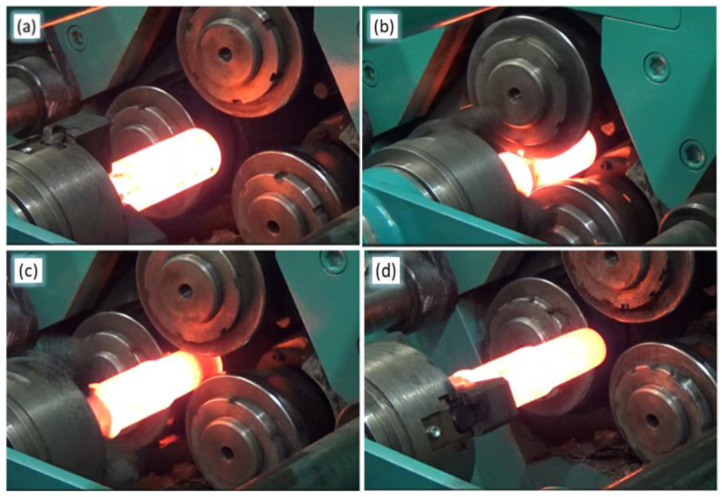
Steps in the forming of bimetallic material in a CNC skew rolling mill: (**a**) billet fixed in the chuck; (**b**) beginning of rolling; (**c**) forming the second step of the workpiece; (**d**) formed bimetallic product.

The different steps in the rolling process of the bimetallic material are shown in Figure 6. The heated billet is placed in the chuck and then moved into the workspace of the rolling mill (Figure 6a). Then, as a result of the execution of the control program entered into the controller, a sequence of tool and chuck movements is executed allowing the production of a bimetallic forging with the assumed geometric dimensions. During the radial movement of the rollers in the direction of the rolling axis, the tool penetrates the workpiece with a preset diameter reduction, this contributes to a change in the cross-section of the rolled forging. In contrast, the rotary movement of the rolling material is the result of tool contact with the rolling workpiece (Figure 6b). At the same time, the chuck moves at a preset velocity along the rolling axis, providing controlled axial rolling motion (Figure 6c). Once the bimetallic forging has exited the workspace, all movements of the CNC rolling mill are stopped in order to pull it out of the chuck (Figure 6d).

## 4. Results

**Figure 7:**
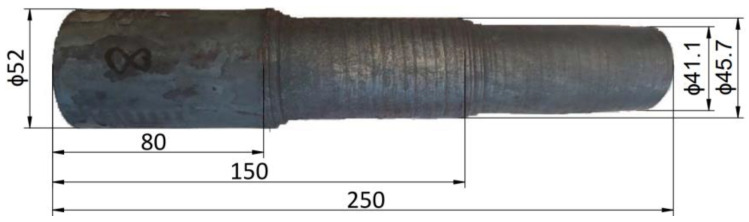
Example of a bimetallic forging produced in a CNC skew rolling mill.

The result of the tests carried out was the formation of a series of stepped forgings with the assumed contour (Figure 7).

**Figure 8:**
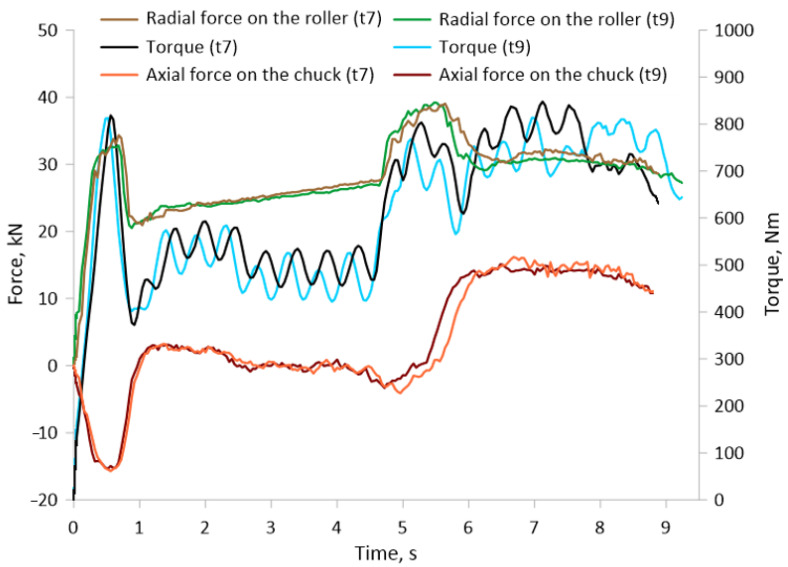
Characteristics of the force course during the rolling of a bimetallic billet with an outer layer thickness (tube) of t = 7 and t = 9.

During the experiments, the force parameters of the process were measured, such as torques, radial forces acting on the tools and the axial force on the chuck. Figure 8 shows a graph of the radial and axial force and torque measured when rolling bimetallic material at initial outer layer thicknesses t = 7 mm and t = 9 mm. No significant differences in the course of radial force, axial force and torque between the different initial thicknesses (t) of the outer layer of the bimetallic component are noted. The course of the radial force is characteristic of this type of rolling of a two-step element. In the initial stage up to about 0.8 s, there was a sudden increase in force, which was related to the penetration of the tools into the workpiece. During the rolling of the first step of the forging (0.8–4.8 s), where the diameter reduction value (δ) was smaller, the force stabilized and was about 25 kN. In the subsequent stages of the rolling process (4.8–5.6 s), the force increased again due to the penetration of the tools into the material up to the preset diameter on the second step. Once the tools had penetrated the material to the preset diameter at the second forging step, the force value stabilized and was approximately 30 kN. In the final rolling stage, the force value decreased slightly. The course of the torque function is similar to the radial force. At the first rolling step, the torque was approximately 500–550 Nm. On the other hand, at the second rolling step of the forging, where the diameter reduction was greater, it was approximately 700–750 Nm. The course of the axial force generated at the chuck had a slightly different character. In the initial stage of the process, as the rollers penetrated the material, the force took on negative values. This means that the chuck was being pushed by the workpiece. This was due to the dependence of the circumferential speed of the tools, which were skewed by an angle of ϴ = 5°. During the forming of the first step when the chuck was moving at a preset velocity of 15 mm/min, the axial force fluctuated around zero. This means that the axial velocity of the chuck was close to the component of velocity in the axial direction generated by the tools during rolling. On the other hand, at the second step, the set velocity on the chuck was higher, at Vc = 25 mm/min, and therefore the axial force also took on positive values of about 15 kN. For skew rolling, it is important to select a suitable axial rolling velocity so that the axial force is near zero or positive. In this way, the selected velocities mean that the entire rolling process is controlled by the set axial velocity at the chuck. This reduces the danger of buckling of the rolling material.

**Figure 9:**
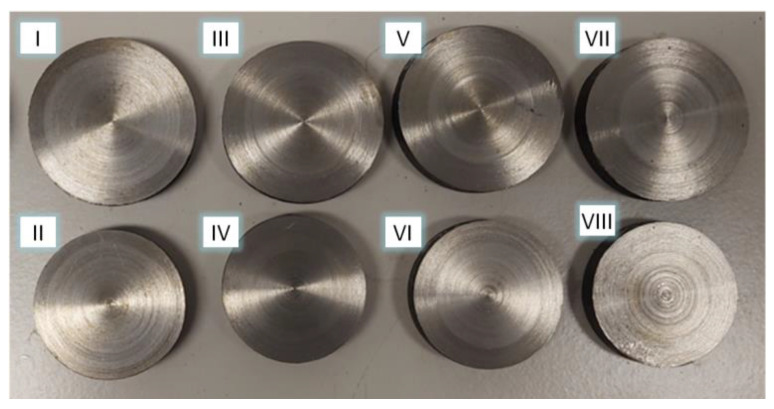
Macroscopic cross-sectional view of the bimetals obtained (variants I–VIII).

The bimetal forgings produced were cleaned and visually inspected. Sections were then taken and analyzed macroscopically to determine the quality of the weld (Figure 9). Analysis of the cross-sections showed that in all eight variants, no clear separation of the joined surfaces could be observed. This means that in all variants, there was a permanent welding of two different materials around the perimeter.

**Figure 10:**
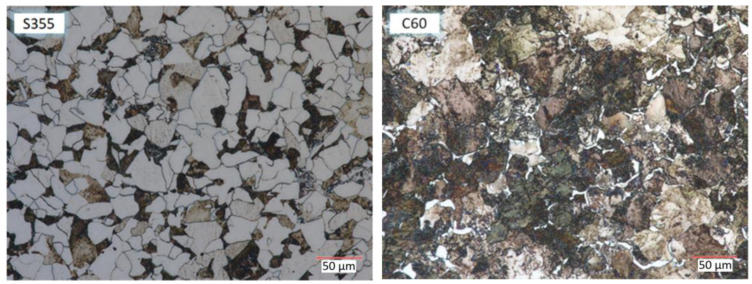
Microstructure of the billet.

**Figure 11:**
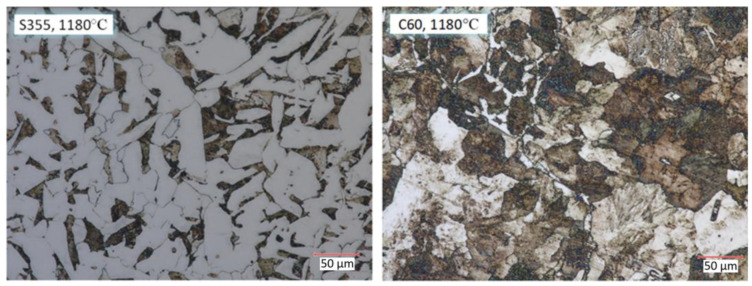
Microstructure of material taken from the chuck zone of a bimetallic forging rolled at 1180 °C.

**Figure 12:**
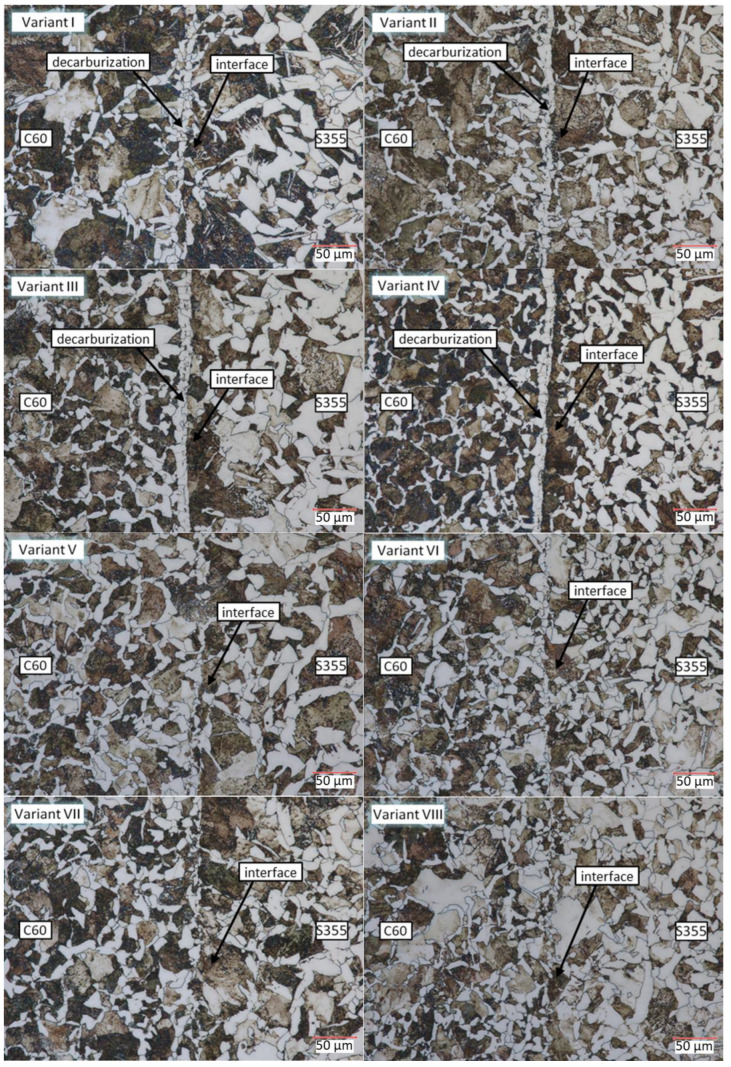
Microstructure in the interface zone of two materials, C60 and S355 (variants I to VIII).

For microscopic analysis of the resulting bimetals, samples were prepared for analysis from the billet and the resulting bimetallic materials (Figure 10, Figure 11 and Figure 12). Metallographic observation was carried out at 700× magnification.

Figure 10 shows the microstructure of the billet of S355 and C60 steels. The microstructure of S355 steel is characterized by a homogeneous structure with an equilibrium mixture of ferrite and pearlite. The proportion of pearlite in S355 steel is about 27%. Microstructural analysis of C60 steel shows a heterogeneous pearlite microstructure with separations of ferrite. The percentage of pearlite in C60 steel is 87%.

The microstructure taken from the chuck zone of a bimetallic forging in which there was no metal forming process is shown in Figure 11. A significant effect of temperature on grain size is observed. In both S355 and C60 steel, there was grain growth under temperature (1180 °C).

Figure 12 shows the microstructure taken from the bimetallic forging in the interface zone between the two materials (variants I to VIII) with the characteristic microstructure zones marked. In all variants, a heterogeneous pearlitic–ferritic structure is visible. In C60 steel, pearlite is present with separations of ferrite along the grain boundaries. The pearlite percentage is 82%. In S355 steel, the proportion of ferrite predominates and traces of Widmanstätten structures are visible. In the variants whose front surfaces were not protected by fusion welding (variants I–IV), a clear joint interface consisting mainly of ferrite grains is visible. This is caused by decarburization of the joined layers during heating in the chamber furnace due to contact with the furnace atmosphere. In the following variants (V–VIII), no visible joint interface is observed. A gentle mixing of the ferrite and pearlite grains has taken place. The microscopic analysis carried out confirms the negative effect on the microstructure of heating in the chamber furnace without adequate protection against oxidation and decarburization.

At this stage of the research, it was not possible to use another type of heating method that would guarantee the reduction of oxidation and decarburization of the material. At this stage of the research, there was no possibility of using another heating method that would guarantee the reduction of oxidation and decarburization of the material. However, research is being conducted to reconstruct the heating system to enable the material to be heated in an atmosphere of noble gases (argon). The use of such a heating method will result in a better-quality joint, which will enable higher shear strength values for the bimetallic material.

**Figure 13:**
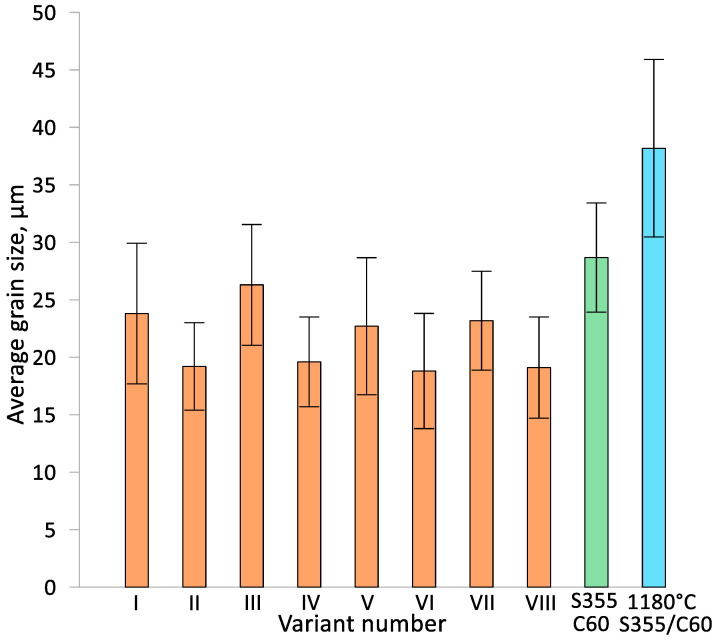
Average grain size in the joining zone of the two materials (variants I–VIII) in relation to the average grain size of the billet and the material taken from the chuck zone of the bimetallic forging.

Measurements were taken of the average grain size in the weld zone of the two materials, the billet and the material taken from the chuck zone without metal forming and previously heated to a rolling temperature of 1180 °C. The results obtained are shown in Figure 13. In all variants of the bimetallic rolling process (I–VIII), the effect of metal forming on the grain size is noticeable in relation to the material taken from the chuck zone (without metal forming). This means that the grain size subjected to metal forming (rolling) is smaller than without forming. In all process variants, there is a significant value of the standard deviation of the mean grain size distribution indicating heterogeneity of the microstructure. The effect of diameter reduction values on grain size is noticeable. At larger diameter reductions, i.e., δ = 1.27 (variant II, IV, VI and VIII), the average grain size is smaller than in the other variants, where a smaller diameter reduction of δ = 1.14 was used, by about 5–6 μm and is about 18–19 μm. No effect of pre-welding on billet fronts (variants V–VIII) on the difference in grain size in the bimetallic forging with respect to rolling from billets without this type of protection (variant I–IV) is noted. A slight effect of the initial outer layer thickness t (t = 7 mm and t = 9 mm) on the average grain size is noticeable. The average grain size is slightly higher for the use of an initial wall thickness t = 9 mm. This can be explained by the fact that plastic strain decreases in the direction of the forming axis.

**Figure 14:**
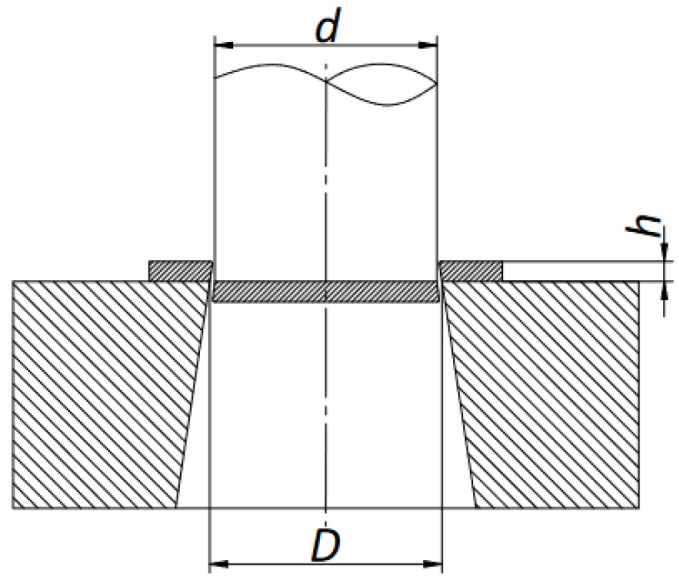
Bimetal shear diagram; h = 10 mm, D = d + 2 mm.

In order to determine the quality of the resulting weld, strength tests were carried out using the shear method according to the scheme shown in Figure 14. The thickness of the material to be sheared was taken as h (h = 10 mm). The diameter of the hole (D) was chosen to be 2 mm larger than the punch diameter (d).

**Figure 15:**
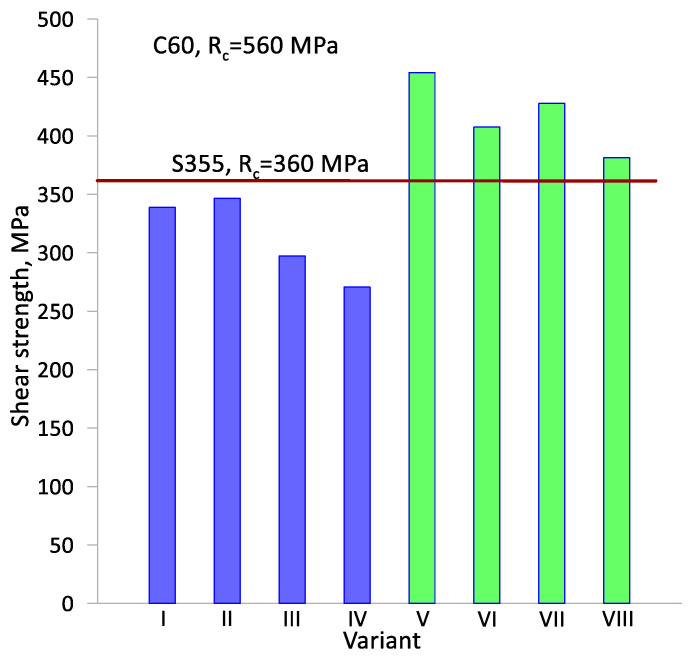
Graph of the shear strength (Rc) of the bimetals obtained during skew rolling in a CNC rolling mill in relation to the Rc strength of homogeneous materials.

The obtained test results for the shear strength (R_c_) of the resulting weld between the two materials in relation to the strength of the homogeneous materials are shown in Figure 15. The resulting weld between the two materials can be considered correct if the weld strength of the two materials exceeds the R_c_ strength of the weaker homogeneous material used. However, traditional fusion welding in some weld schemes assumes a weld strength that is less than that of the weaker material, even by about 20%. The shear strength of the homogeneous materials measured in the same way as the bimetal strength is 360 MPa for S355 steel and 560 MPa for C60 steel. Analyzing the obtained shear strength values, only in the last four variants (variants V–VIII) is the strength higher than the weaker homogeneous material S355. In the initial four variants (variants I–IV), the strength R_c_ is close to the strength of the homogeneous material but does not exceed the limiting value of R_c_ = 360 MPa. This indicates a significant effect of the way the billet material is heated on the strength of the resulting weld. In variants where the billet front surfaces have been protected against decarburization and oxidation, the strength R_c_ is higher. The influence of the diameter reduction value (δ) on the strength can be seen. At higher values of diameter reduction δ = 1.27 (variants IV, VI and VIII), the strength of Rc is lower by about 40 MPa. Only in variant II is the shear strength close to the smaller diameter reduction δ = 1.14. The effect of the initial billet outer layer thickness (t) is also noticeable. For a smaller outer layer thickness t = 7 mm, the strength Rc is higher by about 50 MPa. This is explained by the occurrence of greater plastic strain (pressure on the layers to be joined) during rolling in the near-surface zones of the component.

## 5. FEM Analysis

**Figure 16:**
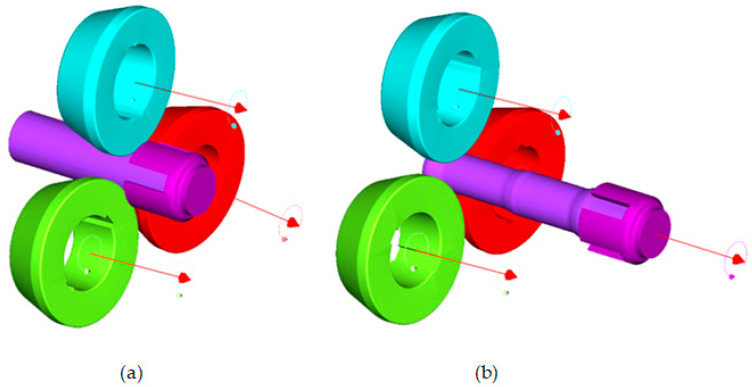
Geometric model used during numerical simulations of bimetallic rolling: (**a**) beginning of rolling process; (**b**) end of rolling process.

Numerical simulations using finite elements analysis (FEM) were carried out in Simufact Forming 21 software. Two numerical simulations were performed, changing the dimensions of the billets—the diameters of the rods and tubes were assumed as in variants V–VIII. The other conditions were unchanged. All parameters were the same as in the experimental tests. Figure 16 shows the geometric model of the process. For numerical simulations, geometric models were made of three identical rollers, a chuck and a billet—a tube with a rod inside it. The length of the billet was 190 mm, the rods were characterized by diameters of ϕ34 mm and ϕ38 mm (depending on the wall thickness of the tube t). The outside diameters of the tubes were ϕ52 mm. The material models were taken from the Simufact Forming (v.15, MSC Software Company, Hamburg, Germany) software’s material library. For a tube made of C60 steel, the material model equation is described by Equation (2), while for a rod made of S355 steel, Equation (3) applies.

(2) $$\sigma_{F}=3245.96\times e^{-0.0033T}\times \varepsilon^{\left(-0.00001468T-0.0825\right)}{\times e}^{\frac{\left(-0.00003956T+0.0122\right)}{\varepsilon }\times {\dot{\varepsilon }}^{\left(0.0001352T+0.00844\right)}}$$

(3) $$\sigma_{F}=2478.72\times e^{-0.00298T}\times \varepsilon^{\left(-0.000423T+0.3659\right)}\times e^{\frac{\left(-0.0000751T+0.0316\right)}{\varepsilon }\times {\dot{\varepsilon }}^{\left(0.00026T-0.1374\right)}}$$

where T−temperature, ε−strain(effective), and $\dot{\varepsilon }-strainrate$.

For the numerical simulations, the rod and tube were assumed to deform, while the other elements—the rollers and the chuck—were established as rigid objects. The tube and rod were divided into first-order cubic elements with an element size of 1.75 mm for the tube, which gave a total of 31.000 elements and 1.50 mm for the rod, making it 21.000 elements. The initial temperature of the billet was set at 1180 °C, the temperature of the shaping tools (rollers) was set at 100 °C and the chuck temperature was set at 250 °C. The heat transfer coefficient between the billet and the forming rollers was assumed to be 10 kW/m^2^K and the billet-environment heat transfer coefficient was set to 0.50 kW/m^2^K. These coefficients were taken from the Simufact Forming database. A constant friction model was adopted for the analyzed process, which is described by Equation (4). The friction coefficient between the rollers and the billet m = 0.95, between the rod and the tube m = 1 and between the billet and the chuck the friction coefficient m = 1.

(4) τ=mk

where τ−shear stress on contact surface, m−friction coefficient and k−shear yield stress. 

In addition, a simplification was adopted that assumed that the rod and the tube were glued for simulation purposes. The tools were moving: the rollers rotated in the same direction at the same speed of 60 rpm, and in order to shape the billet, the tools performed radial movement in the direction of the axis of the shaped bimetallic rod at the same speeds as during the experimental tests.

### 5.1. Numerical Model Verification (FEM)

**Figure 17:**
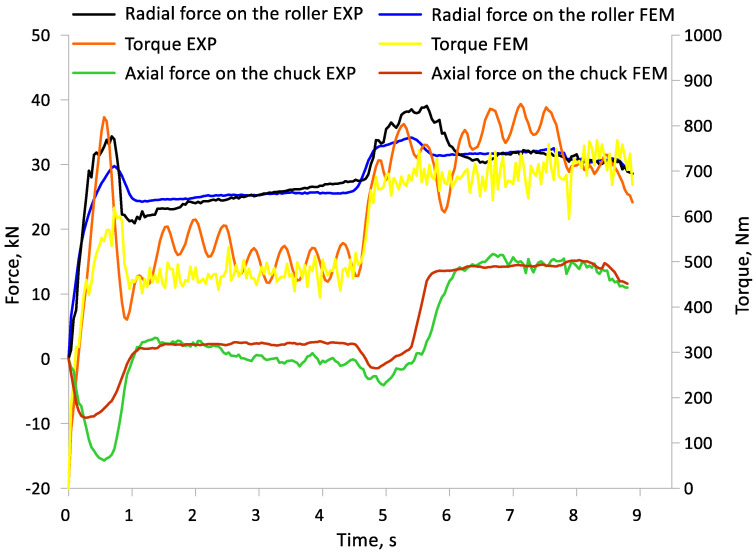
Force comparison characteristics from experimental tests and FEM analysis during forming of a bimetal by rolling.

The numerical model of the skew rolling process of bimetallic parts in the Simufact Forming (v.15, MSC Software Company, Hamburg, Germany) software was verified by comparing the course of forces obtained in FEM and experimental tests (Figure 17). Due to the similar values of forces and torque obtained during the experimental tests between the initial outer layer thicknesses (t), the course of forces recorded during the rolling of a billet with an outer layer thickness of t = 7 mm was selected for analysis. The quantitative agreement of the force course was measured with the coefficient of determination R^2^. The highest quantitative concordance was obtained in the case of the axial force, where the coefficient of determination R^2^ is 0.92. This shows a very good match between the axial force characteristics obtained from the tests and the axial force obtained from FEM. In the case of the radial force, the R^2^ coefficient is 0.82. This type of value obtained indicates good agreement of the numerical model. The lowest R^2^ value was obtained for a torque of 0.77. This is related to the slightly higher torque obtained during the experimental tests. The reason for this is the resulting resistance on the transmission elements, which was not taken into account in the numerical simulation. However, in spite of this, the obtained value of the coefficient of determination for the torque can be considered to indicate satisfactory agreement.

**Figure 18:**
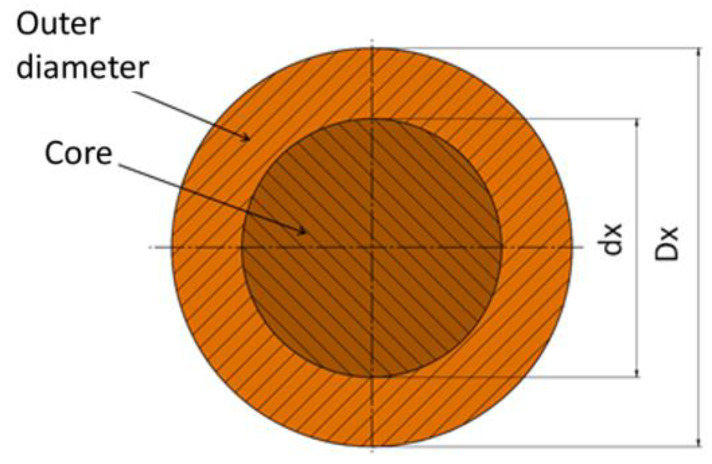
Measurement indications for bimetal forging diameters.

In addition, the numerical model was verified geometrically by measuring the individual diameters of the bimetallic forging (Figure 18).

The dimensions obtained after the experimental tests in comparison with the dimensions obtained from the numerical simulation (FEM) are shown in Table 3. A high dimensional agreement was observed between the results from the diameters obtained from the experimental tests and the measurements from the FEM. Thus, the numerical model (FEM) of skew rolling of bimetallic can be considered good.

### 5.2. Analysis of Temperature Distribution

**Figure 19:**
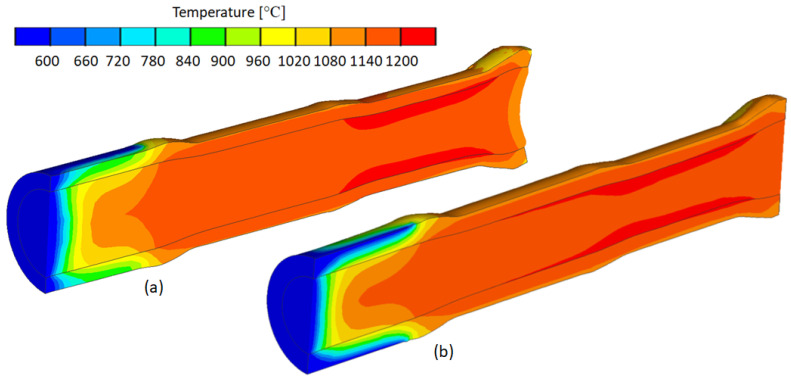
Temperature distribution in the range of (600–1200) °C: (**a**) thickness of outer layer of billet t = 7 mm; (**b**) thickness of outer layer of billet t = 9 mm.

As a result of the numerical simulations, the distributions of temperature, effective plastic strain, circumferential stresses, radial stresses and equivalent stresses were determined. The results of the numerical analysis confirm the forming capability of the bimetals. Figure 19a shows the temperature distribution for a bimetal with outer layer thickness t = 7 mm, while Figure 19b shows the rolling temperature distribution for a bimetal with outer layer thickness t = 9 mm. The temperature at which the bimetal is rolling has a strong influence on the quality of the resulting weld due to the diffusion bonding character of the two materials. It is desirable to roll at high temperatures. The greatest drop in temperature of the forming product becomes apparent in the chuck zone as a result of contact between the hot product and the chuck at a temperature of 250 °C. In the layers directly in contact with the chuck and near-surface, the temperature oscillates in the range of (600–660) °C. During the rolling of the bimetal, cooling occurs in the near-surface layer due to contact with the forming cold rollers and as a result of convection and radiation. However, the temperature drop is insignificant, the rolling component still being in the hot forming range of the steel. This does not affect the temperature value at the joining point of the two materials in any way, as the temperature in the zone of the materials to be welded is in the range of (1140–1200) °C.

### 5.3. Strain Analysis

**Figure 20:**
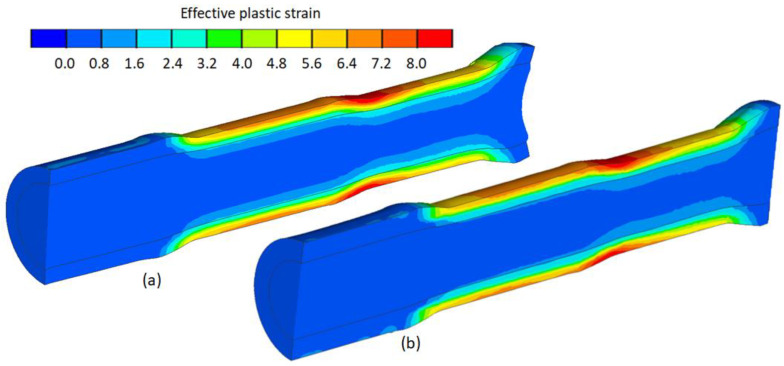
Effective plastic strain distribution: (**a**) thickness of outer layer of billet t = 7 mm; (**b**) thickness of outer layer of billet t = 9 mm.

In the case of rolling bimetallic rods, the influence of metal flow kinematics as well as strain distribution on the quality of the resulting material weld is important. During skew rolling, it is characteristic that the material flows more intensively in the surface and near-surface layers. As a result, the outer layer (t) is reduced. Figure 20 shows the differences in the effective plastic strain of a rolled bimetallic component. The largest deformations are in the outer layer (as a result of contact with forming tools) and decrease towards the bimetal axis. It can be concluded that at the welding of the thinner tube (t = 7 mm) with the rod, the value of strain is higher (Figure 20a). Therefore, the quality of the weld should be better than for a tube with a larger thickness (t = 9 mm), whose strain distribution is shown in Figure 20b. On the second stage of the rolled bimetallic rod, the strain is to a higher depth due to the higher reduction ratio (δ).

### 5.4. Stress Analysis

**Figure 21:**
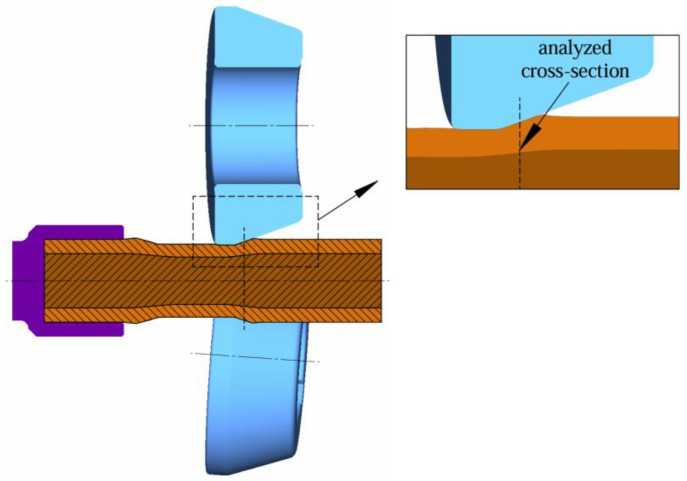
Schematic of the skew rolling bimetallic rod with the section marked that was subjected to stress analysis during rolling.

Another extremely important aspect is the condition of stress during rolling, which has an important role in the case of diffusion bonding. Compressive radial and circumferential stresses in the material welding zone facilitate the diffusion bonding of two materials. This means that enough pressure is exerted for diffusion to take place and the materials being joined remain in continuous contact. Radial stresses, circumferential stresses and reduced stresses were analyzed in the forming zone. Figure 21 shows a rolling diagram with a marked cross-section on the first formed stage, which was subjected to stress analysis. For the second stage (with a larger diameter reduction), the cross-sections were made in the same way.

**Figure 22:**
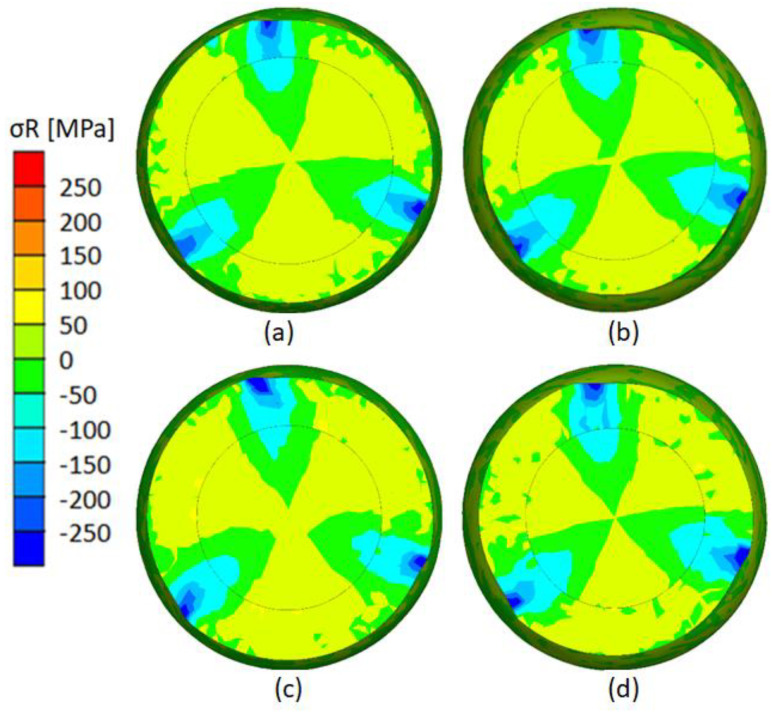
Radial stress distribution: (**a**) variant V (t = 7 mm, δ = 1.14); (**b**) variant VI (t = 7 mm, δ = 1.27); (**c**) variant VII (t = 9 mm, δ = 1.14); (**d**) variant VIII (t = 7 mm, δ = 1.27).

Based on the obtained distribution of radial stresses (Figure 22) during rolling, the occurrence of compressive stresses can be noticed. The areas with the highest values of these stresses are located in the forming zones at the contact zones between the forming rollers and the rolled billet. It is during this rolling stage that a potentially permanent bond between two materials is formed. In the welding zone for variants V and VI, where the outer layer thickness (t) was 7 mm (Figure 22a,b), the radial compressive stresses were higher by about 50 MPa than in variants VII and VIII, where the outer layer thickness (t) was 9 mm (Figure 22c,d). Therefore, the further into the material, the stresses decrease. This limits the potential use of very thick outer layers of bimetallic material, due to the low compressive radial stresses there. The location of these stresses changes during forming, which is due to the kinematics of the process. In other areas not in the forming zone, these stresses have values close to zero or positive but relatively low. This can be disadvantageous in terms of potential welding, because it promotes separation of the surfaces to be welded. However, as experimental tests show, the resulting combination of two materials when the roll comes into contact with the rolled element is strong enough not to cause separation of the materials being welded.

**Figure 23:**
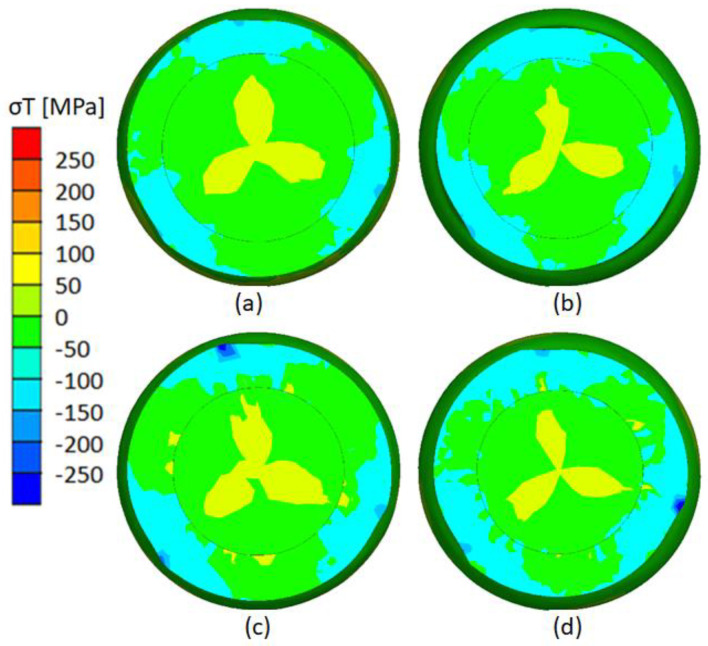
Circumferential stress distribution: (**a**) variant V (t = 7 mm, δ = 1.14); (**b**) variant VI (t = 7 mm, δ = 1.27); (**c**) variant VII (t = 9 mm, δ = 1.14); (**d**) variant VIII (t = 7 mm, δ = 1.27).

The process of welding two different materials during skew rolling is strongly influenced by the condition of circumferential stresses (Figure 23). Analyzing the distribution of circumferential stresses, one can see the occurrence of compressive stresses appearing in areas along the entire circumference of the rolled element. These stresses also extend to the weld zone between the tube and the rod. This is very advantageous because it contributes to the continuous adhesion of the surfaces to be welded together, which allows a permanent combination to be formed. The highest values of compressive circumferential stresses are noticeable in the areas of contact between the rollers and the rolled element and are on average 50 MPa higher than outside the forming zone (contact between the tools and the billet). Analyzing the influence of outer layer thickness (t), it is noted that larger compressive circumferential stresses occur over a larger area in the weld zone for smaller outer layer thickness (Figure 23a,b). Therefore, this distribution of circumferential stresses is less favorable for rolling bimetallic rods with larger wall thicknesses (Figure 23c,d). No significant effect of diameter reduction (δ) on the value of circumferential stresses can be seen.

**Figure 24:**
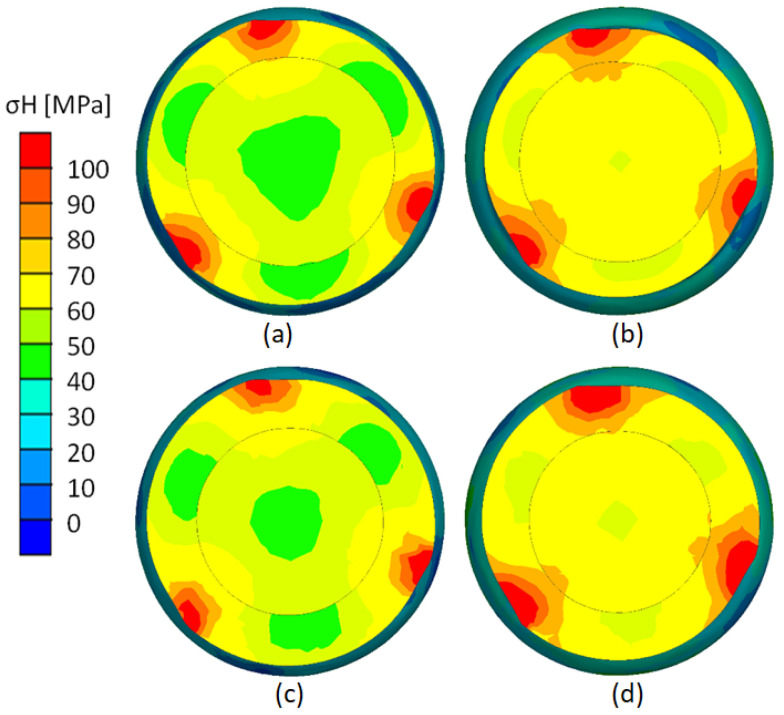
Reduced stress distribution: (**a**) variant V (t = 7 mm, δ = 1.14); (**b**) variant VI (t = 7 mm, δ = 1.27); (**c**) variant VII (t = 9 mm, δ = 1.14); (**d**) variant VIII (t = 7 mm, δ = 1.27).

Figure 24 shows the distribution of reduced stresses according to the Huber–Mises hypothesis. Reduced stresses reach their highest values in the contact zone between the rollers and the bimetallic rod. These areas displace during rolling due to the kinematics of the process. On the second stage of the billet, these stresses are larger. The closer to the axis of the bimetallic rod, the more uniform the reduced stresses.

## 6. Conclusions

As a result of the experimental and theoretical tests carried out by the finite element method (FEM) on rolling bimetallic elements of C60 and S355 steel, it is concluded that it is possible to produce this type of material by skew rolling technology in an innovative CNC skew rolling mill.

The obtained experimental test results lead to the following conclusions:

- There is a strong influence of the heating method on the microstructure in the weld zone. In the variants in which the billets were heated in a chamber furnace without a protective atmosphere, there is a clear boundary between the two materials consisting mainly of ferrite grains at the weld interface.
- In the variants where the billets were protected from decarburization and oxidation phenomena in the microstructure, no clear boundary of the weld is noticed.
- The microstructure at the junction of two materials is characterized by inhomogeneity (heat treatment is recommended to homogenize the microstructure).
- The 1180 °C billet heating temperature significantly affects the growth of the average grain size. However, as a result of metal forming by rolling, the average grain size is reduced to a grain size close to the grain size of the billet (before heating).
- A larger value of diameter reduction during bimetal rolling (δ = 1.27) results in a smaller average grain size by about 5–6 μm compared to the smaller value of diameter reduction (δ = 1.14).
- No significant influence of the heating method was observed in the temperature range of (600–1200) °C (billets fusion-welded on the fronts and not fusion-welded) on the average grain size.
- Better shear strength values exceeding the strength of the weaker homogeneous metal S355 used were achieved for variants in which the billets were protected from oxidation and decarburization before rolling.
- Rolling bimetallic materials with a thinner outer layer results in better shear strength.
- Rolling with a higher reduction in diameter contributes to lower shear strength.

The obtained theoretical results lead to the following conclusions:

- The rolling temperature of the bimetal in the welding zone of the two materials, despite contact with cold tools and convection and radiation, is within the assumed range of hot forming of steel.
- The distribution of deformation is more intense in the near-surface layers of the rolled bimetal and when rolled with larger reductions in billet diameter.
- Compressive radial stresses occur in the zone of contact between the tools and the rolling bimetal, and tensile stresses occur outside this zone. The character of these stresses is variable due to the kinematics of the process. This can cause difficulties in welding the two materials together.
- There are compressive circumferential stresses throughout the welding zone of the two materials. This causes continuous adhesion of the surfaces to be joined to each other, making it much easier to achieve a permanent weld.
- Higher values of compressive stresses are present in cases of bimetal rolling in which the thickness of the outer layer of the billet is smaller.

The obtained research results indicate that the proposed technology enables efficient production of both bimetallic rods with a constant cross-section and axisymmetric bimetallic forgings. At present, there are no efficient methods for manufacturing bimetallic rods, which, thanks to their properties, can find applications in various areas of the economy. The proposed solution is a response to the needs of the forging industry in this area. Therefore, it is advisable to continue research in this field, which will be aimed at the industrial implementation of skew rolling methods for bimetallic rods.

## Figures and Tables

**Table 1:** Chemical composition of C60 and S355 steels.

Material	C	Si	P	S	Cr	Mo	Ni
C60	0.57–0.65	0.40	0.045	0.045	0.40	0.10	0.40
S355J	0.22	0.55	0.04	0.04	0.29	0.11	0.04

**Table 2:** Input factors for the rolling process of the bimetallic components.

Billet	Variant	D, mm	d, mm	t, mm	*δ* = D/D_x_	Vc, mm/s	Welded Fronts
1	I	52	38	7	1.14	15	no
	II	52	38	7	1.27	25	no
2	III	52	34	9	1.14	15	no
	IV	52	34	9	1.27	25	no
3	V	52	38	7	1.14	15	yes
	VI	52	38	7	1.27	25	yes
4	VII	52	34	9	1.14	15	yes
	VIII	52	34	9	1.27	25	yes

**Table 3:** Comparison of measurements of individual diameters from experimental tests and from numerical modeling (FEM).

Thickness of Outer Layer of Billet (t) 7 mm				
Type of test	Diameter reduction δ = 1.14		Diameter reduction δ = 1.27	
	Outer diameter D_x_, mm	Core diameter d_x_, mm	Outer diameter D_x_, mm,	Core diameter d_x_, mm
Experiment	45.7	33.8	41.1	30.8
FEM	45.4	33.2	40.9	30.4
Thickness of Outer Layer of Billet (t) 9 mm				
Type of test	Diameter reduction δ = 1.14		Diameter reduction δ = 1.27	
	Outer diameter Dx, mm	Core diameter dx, mm	Outer diameter Dx, mm,	Core diameter dx, mm
Experiment	45.7	31.1	41.0	27.8
FEM	45.4	30.6	40.9	27.2

## Data Availability

The data presented in this study are available on request from the corresponding author.
